# Neutralizing immune responses induced by oligomeric H5N1-hemagglutinins from plants

**DOI:** 10.1186/s13567-017-0458-x

**Published:** 2017-09-20

**Authors:** Hoang Trong Phan, Thuong Thi Ho, Ha Hoang Chu, Trang Huyen Vu, Ulrike Gresch, Udo Conrad

**Affiliations:** 1 Leibniz Institute of Plant Genetics and Crop Plant Research (IPK), Gatersleben, Germany; 2 Institute of Biotechnology, Hanoi, Vietnam

## Abstract

Plant-based transient expression is an alternative platform to produce hemagglutinin-based subunit vaccines. This production system provides not only fast and effective response in the context of a pandemic but also enables the supply of big volume vaccines at low cost. Crude plant extracts containing influenza hemagglutinin are considered to use as vaccine sources because of avoidance of related purification steps resulting in low cost production allowing veterinary applications. Highly immunogenic influenza hemagglutinins are urgently required to meet these pre-conditions. Here, we present a new and innovative way to generate functional H5 oligomers from avian flu hemagglutinin in planta by the specific interaction of S·Tag and S·Protein. A S·Tag was fused to H5 trimers and this construct was transiently co-expressed in planta with S·Protein-TPs which was multimerized by disulfide bonds via cysteine residues in tailpiece sequences (TP) of IgM antibody. Multimerized S·Protein-TPs serve as bridges/molecular docks to combine S·Tag-fused hemagglutinin trimers to form very large hemagglutinin H5 oligomers. H5 oligomers in the plant crude extract were highly active in hemagglutination resulting in high titers. Immunization of mice with two doses of plant crude extracts containing H5 oligomers after storage for 1 week at 4 °C caused strong immune responses and induced neutralizing specific humoral immune responses in mice. These results allow for the development of cheap influenza vaccines for veterinary application in future.

**Electronic supplementary material:**

The online version of this article (doi:10.1186/s13567-017-0458-x) contains supplementary material, which is available to authorized users.

## Introduction

Influenza A viruses, negative-stranded enveloped orthomyxoviruses, are among the most serious respiratory pathogens. They cause severe and potentially fatal illnesses [1]. Highly pathogenic avian flu influenza viruses are expected to cause the next global pandemic threat due to their relative easy spread by avian hosts and their ability to directly infect humans [2]. During several H5N1 outbreaks, very large direct and indirect impacts on poultry and tourist industries in South-East Asia were observed [3]. Recently, highly pathogenic avian influenza viruses (HPAI) A (H5N8) caused outbreaks in South Korea, China and Japan and were actually reported in many European countries as well as in the US and Canada (for an review, see [4]). Therefore, the development of an effective and cheap vaccination strategy to protect poultry is necessary. This should include high efficacy of the vaccine, easy and sure production strategies and an efficient way to handle distribution and application. Here, subunit vaccines from plants, which have the general advantages of low production cost, ease of scale up, low infrastructure cost, high stability and long shelf life are in the focus [5]. Transient expression in tobacco plants has been developed as a very fast and efficient method to produce therapeutic proteins in plants (for a review, see [6]). A recently developed strategy is the production of virus-like particle (VLP)-based vaccines in the tobacco species *N. benthamiana*, by transient expression and downstream processing steps that include several filtrations, diafiltrations, continuous flow centrifugations and tangential flow filtration, or, alternatively, chromatographic methods [7, 8]. The production of VLP’s is accompanied by several constraints such as high down-stream cost and/or low expression levels. We produced trimeric H5 hemagglutinins in the endoplasmic reticulum of plant leaf cells. An artificially designed trimerization domain (GCN4-pII, [9]) was used to achieve stable trimers of H5 hemagglutinins from plants [10]. The purified trimers were shown to be active in a hemagglutination assay and also induced neutralizing humoral immune responses as shown by mouse immunization and hemagglutination inhibition assays. We also developed a suitable cheap and efficient purification system from plant extracts by using ELPylation [11].

In the current article we wanted to check, if a further increase of the size of H5 multimers could improve the induction of immune responses. Because hemagglutinin forms trimers in its’ native structure on the surface of viruses we planned to keep the trimers as a basic structure of the oligomers. Further oligomerization should be caused by S·Tag–S·Protein interaction. Bovine pancreatic ribonuclease A, 124 amino acids in length, is cleaved by the protease subtilisin. The cleavage product consists of two tightly associated fragments: S-peptide (residues 1–20) and S·Protein (residues 21–124). Only residues 1–15 of S-peptide were found to be necessary to complex specifically with S·Protein. This shorter fragment is named as “S15” or the “S·Tag” sequence [12]. High-affinity interaction between S·Protein and S·Tag of bovine pancreatic ribonuclease A was recently developed as target for protein purification [12] or for drug delivery [13]. We applied this technology to generate H5 oligomers. To achieve this goal, a S·Tag was fused to H5 trimers and this construct was transiently co-expressed with different multimeric variants of S·Protein in planta.

**Figure 1:**
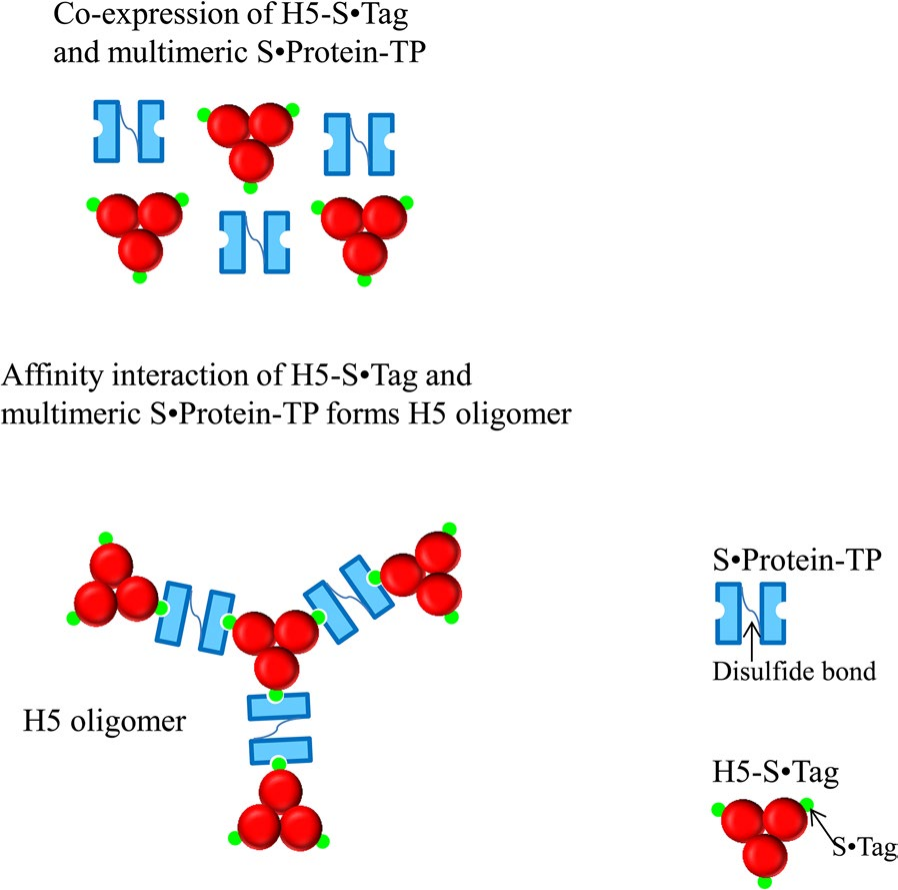
**Model of H5 oligomer formation by co-expression of H5-S·Tag and multimeric S·Protein-TP oligomerized by disulfide bonds.** The oligomeric state of the S·Protein-TP depends on the oligomeric state of the wild-type S·Protein which is a mixture of the dominant monomer, as well as minor dimers, trimers, etc [23]. Fusion of wild-type S·Protein to TP causes additional linkage via disulfide bonds to generate multiple S·Proteins. S·Protein-TP, depicted here as an example, is a homodimer formed by a disulfide bond.

These variants are based on different structures that support the multimerization of S·Protein, as GCN4 (dimerization), GCN4-pII (trimerization) [9] and tailpiece (TP) from IgM [14] (dimerization, Figure 1). Multimerized S·Proteins serve now as bridges/molecular docks to combine S·Tag-fused hemagglutinin trimers to form hemagglutinin oligomers (Figure 1). Furthermore, we wanted to proof, if neutralizing immune responses could also be achieved by immunization with plant crude extracts, thus minimizing the down-stream cost. Enlargement of immunogenic trimers to oligomers was successfully proven to enhance immunogenicity by inducing neutralizing antibodies. Here we showed that crude plant extracts containing H5 oligomers could induce specific and strong immune responses against H5 hemagglutinin in mice.

## Materials and methods

### Construction of plant expression vectors

**Figure 2:**
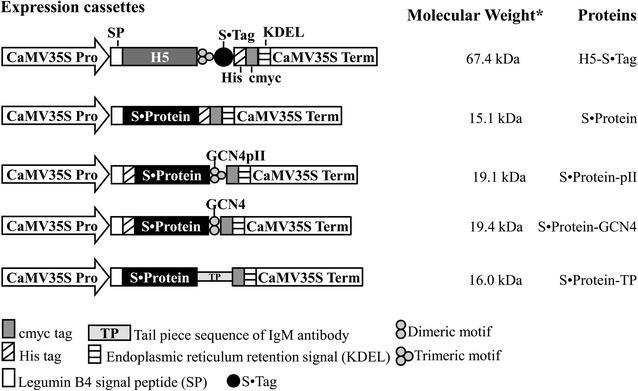
**Expression cassettes for the in planta production of H5-S·Tag and S·Protein fusion proteins.** The ectodomain of hemagglutinin (H5) was trimerized by c-terminal fusion of trimeric motif (GCN4pII) [10]. While wild-type S·Protein was either expressed alone or multimerized by fusion with different oligomeric motifs, such as trimerization (GCN4-pII, S·Protein-pII) and dimerization (GCN4 wild-type, S·Protein-GCN4) domains [9], and a mouse tailpiece (TP) element of mouse IgM (S·Protein-TP). Each of recombinant proteins was fused to a c-myc tag to allow for downstream detection by Western blot, a His tag (except for S·Protein-TP) to facilitate their purification by IMAC. The legumine B4 signal peptide and the KDEL motif were used to promote transgene products retention in the endoplasmic reticulum. CaMV35S Pro: cauliflower mosaic virus 35S ubiquitous promoter; CaMV 35S Term: cauliflower mosaic virus 35S terminator; Asterisk: the molecular weight of proteins was calculated for unglycosylated monomers.

The DNA sequences corresponding to aa 2–564 of the hemagglutinin of A/duck/Viet Nam/TG24-01/2005 (H5N1) strain and aa 21–124 of S·Protein (UniProtKB Accession Numbers: Q14RX0 and P61823, respectively) were synthesized commercially (GENECUST EUROPE, Luxembourg) and provided in pUC57 vectors designated pUC57-H5TG and pUC57-S·Protein. To express the wild-type S·Protein, the DNA sequence coding for S·Protein was cloned into pRTRA-35S-H5pII [10] at the BamHI and NotI sites to form a recombinant vector designated pRTRA-S·Protein. To multimerize S·Protein, DNA sequences coding for S·Protein were introduced into pRTRA vectors, which contain trimerization (GCN4-pII) or dimerization (GCN4 wild-type) domains [9], or a tail piece of mouse IgM antibody that forms disulfide bonds via its cysteine residues; the resulting vectors were pRTRA-His-S·Protein-GCN4pII, pRTRA-His-S·Protein-GCN4wt, and pRTRA-S·Protein-TP, which were used for expression of S·Protein-pII, S·Protein-GCN4, S·Protein-TP, respectively (Figure 2). An S·Tag sequence was inserted into pRTRA-H5TG-GCNpII to produce the pRTRA-H5TG-GCNpII-S·Tag vector, which was used for expression of trimeric H5-S·Tag. Five expression cassettes constructed in pRTRA vectors (pRTRA-S·Protein, pRTRA-His-S·Protein-GCN4wt, pRTRA-His-S·Protein-GCN4pII, pRTRA-S·Protein-TP, pRTRA-H5TG-GCNpII-S·Tag) (Figure 2) were cloned into the pCB301 shuttle vector [15] at *Hin*dIII restriction sites. The pCB301 shuttle vectors were introduced into the *Agrobacterium* pGV2260 strain.

### *Agrobacterium* infiltration

Agrobacterium infiltration for expression of recombinant proteins was described in detail by Phan and Conrad, 2016 [16], and is briefly described here. *Agrobacteria* harboring the shuttle vectors for the expression of recombinant proteins (Figure 2) and the plant vector for expression of HcPro, which is a suppressor of gene silencing that has been found to enhance the expression levels of recombinant proteins in plant cells [17, 18] were pre-cultivated separately in LB medium with 50 µg/mL kanamycin, 50 µg/mL carbenicillin and 50 µg/mL rifampicin overnight at 28 °C and 140 rpm. The precultures were added to a new LB culture containing the appropriate antibiotics. After 24 h of cultivation, bacteria were harvested by centrifugation (4000*g*, 30 min, 4 °C) and resuspended in infiltration buffer (10 mM 2-(N-morpholino) ethanesulfonic acid (MES), 10 mM MgSO4, pH 5.6). *Agrobacteria* harboring the shuttle vector for the expression of recombinant protein and the plant vector for the expression of HcPro were combined and diluted in infiltration buffer to a final OD600 of 1.0. *N. benthamiana* plants (6–8 weeks old) were infiltrated by completely submerging each plant in an *Agrobacterium*-containing cup standing inside a desiccator. Vacuum was applied for 2 min and then quickly released. The plants were then placed in the greenhouse at 21 °C, 16 h light per day. Five days after infiltration, leaf samples were harvested and stored at −80 °C. Two agrobacterial strains were mixed and combined with HcPro strain to co-express H5-S·Tag and S·Protein variants. *Agrobacteria* were then diluted in the infiltration buffer, and were used for vacuum infiltration described above.

### Total soluble protein extraction

Five days after vacuum infiltration of *Agrobacterium*, 20 g of leaf samples were harvested, ground in liquid nitrogen and homogenized in 60 mL of PBS buffer (pH 7.4) using a commercial blender. The extracts were clarified by centrifugation (16 200 *g*, 30 min, 4 °C). Total soluble protein contents of clear plant extracts were determined by Bradford assay [19].

Protein concentrations of all plant extracts were diluted to 3 µg/µL. A H5-S·Tag plant extract was then combined with a single S·Protein variant plant extract in equal volume. The mixtures were rotated at 4 °C for 1 h and used for hemagglutination assay. H5 oligomer or H5-S·Tag in crude plant extracts was semi-quantified by Western blotting. A series of known concentrations of the anti TNFα-nanobody-ELP standard protein [20] was used to construct blot signal intensities. Hemagglutinin contents in the plant crude extracts were determined by comparing their blot signal intensities and blot signal intensities of the standard protein.

### SDS-PAGE and Western blotting

Extracted plant proteins, purified proteins, or an anti TNFα-nanobody-ELP standard protein [20] were separated by reducing SDS-PAGE (10% polyacrylamide) and then electrotransferred to nitrocellulose membranes. The Western blotting procedure was carried out using monoclonal anti-c-myc antibodies following the protocol described by Gahrtz and Conrad [21]. Sheep anti-mouse IgG, horseradish peroxidase-linked whole antibody was used as the secondary antibody (secondary antibodies, GE Healthcare UK limited, Little Chalfont Buckinghamshire, UK) followed by enhanced chemiluminescence-based detection (ECL). A total of 10 ng of the IMAC and SEC purified hemagglutinin was separated by reducing SDS-PAGE (10% polyacrylamide) and electrotransferred to nitrocellulose membranes. To detect H5-specific mouse antibodies, ten mouse sera from each group were mixed, and the membranes were incubated with the respective mixtures (200 times dilution). Specific signals were detected as described above.

### Protein purification by IMAC

Five days after vacuum infiltration of *Agrobacterium*, leaf samples were harvested, frozen in liquid nitrogen and homogenized using a commercial blender. Total proteins were extracted in 50 mM Tris buffer (pH 8.0). The extracts were clarified by centrifugation (75 600 *g*, 30 min, 4 °C) and then filtrated through paper filters. The clarified extracts were mixed with Ni–NTA agarose resin that had been washed twice with water beforehand. After mixing for 30 min at 4 °C, the mixture was added to a chromatography column. Thereafter, the column was extensively washed (50 mM NaH_2_PO_4_, 300 mM NaCl, 30 mM imidazole, pH 8.0). Recombinant proteins were then eluted from the column with elution buffer (50 mM NaH_2_PO_4_, 300 mM NaCl, 125 mM Imidazole, pH 8.0), put into dialysis bags, concentrated in PEG 6000 and dialyzed against PBS.

### Purification of H5 oligomer using *Galanthus nivalis* (GLN)-linked agarose

Frozen leaf samples (40 g) were homogenized in liquid nitrogen. Total soluble proteins were extracted in PBS (137 mM NaCl, 2.7 mM KCl, 10 mM Na_2_HPO_4_, 1.8 mM KH_2_PO_4_, pH 7.5). The extract was centrifuged twice (75 600 *g*, 30 min, 4 °C) and mixed with 10 mL of GLN resin that had been washed twice with water and once with PBS. After mixing at 4 °C for 30 min, the mixture was applied to a chromatography column. Thereafter, the column was washed two times with 30 mL PBS. The recombinant protein was then eluted from the column with 10 mL elution buffer (137 mM NaCl, 2.7 mM KCl, 10 mM Na_2_HPO_4_, 1.8 mM KH_2_PO_4_, 200 mM α-methyl mannoside, pH 7.4). The protein solution was dialyzed against PBS at 4 °C overnight and concentrated using PEG 6000. The protein products were loaded on Superose™ 6 increase 10/300GL.

### Size exclusion chromatography

A total of 34 µg protein/0.5 mL of purified H5 oligomers and H5-S·Tag, respectively, were loaded onto a Superose™ 6 increase 10/300GL column (GE Healthcare). The high molecular weight kit contains standard proteins with molecular weights in the range of 44–2000 kDa, which were loaded onto the column to estimate the molecular weight of the proteins of interest. Five hundred microliters per fraction were collected for hemagglutination test and Western blot analysis.

For ELISA, affinity-purified trimeric hemagglutinin was used as an antigen for the plate coating and was further purified via the Superose™ 6 increase 10/300GL column with starting concentrations of 1.25 mg in 0.5 mL.

### Mouse immunization

The hemagglutinin contents (H5 oligomer and H5-S·Tag) in plant extracts were semi-quantified by Western blotting. Plant extracts containing 100 ng of either H5 oligomers or H5-S·Tag were selected for mouse immunization. In the control groups, a plant extract containing S·Protein-TP and a non-transformed plant extract that had the same amount of total soluble protein as plant extracts containing H5 oligomers and H5-S·Tag were used. All plant extracts were formulated with the Emulsigen^®^-D adjuvant (MVP Technologies, 4805 G Street, Omaha, NE 68117, USA) at 20% final concentration. Seven–nine-week-old male C57/Bl6/J mice (Charles River Laboratories, Research Models and Services, Germany GmbH; twelve per group) were subcutaneously immunized with Emulsigen^®^-D adjuvant-formulated plant extracts at days 0, 14 and 28. One week after the 2^nd^ and 3^rd^ immunizations, mice were bled via the retro-orbital sinus. Mouse sera were collected individually for hemagglutination inhibition and ELISA tests.

### Hemagglutination test and hemagglutination inhibition assay

The hemagglutination test was based on a standard protocol [22]. The dilution that induced complete hemagglutination was defined as one hemagglutination unit (HAU). The HI assay was performed similarly based on a standard procedure [22]. Because of unavailability of the A/duck/Viet Nam/TG24-01/2005 (H5N1) virus in an inactivated form, the heterologous inactivated virus strain rg A/swan/Germany/R65/2006(H5N1) was used for HI assay. The deduced hemagglutinin amino acid sequence similarity of both strains is 96%. A 25 µL aliquot of serum from a single mouse was placed in the first well of a microtiter plate containing 25 µL PBS, and two-fold serial dilutions were made across the row of 8 wells. A 25 µL volume containing 4 HAU of the inactivated rg A/swan/Germany/R65/2006(H5N1) virus was added to the reaction and incubated at 25 °C for 30 min. Then, 25 µL of 1% chicken red blood cells was added, and the plates were incubated at 25 °C for 30 min. The HI titer is presented as the reciprocal of the highest dilution of serum that could completely inhibit hemagglutination.

### Indirect ELISA

Microtiter plates (ImmunoPlateMaxisorp, Nalgen Nunc International, Roskilde, Denmark) were coated with 100 µL of 0.5 µg/mL of IMAC- and SEC-purified hemagglutinin trimers in phage PBS (100 mM NaCl, 32 mM Na_2_HPO_4_, 17 mM Na_2_HPO_4_, pH 7.2) and incubated overnight at room temperature. After blocking with 3% (w/v) bovine serum albumin (BSA), 0.05% (v/v) Tween20 in PBS (PBST) for 2 h, 100 µL of the specific dilution (6 × 10^−4^) was applied and incubated at 25 °C for 1 h. Plates were washed 5 times with PBST, incubated with alkaline phosphatase-conjugated rabbit anti-mouse IgG diluted (2000 times) in 1% (w/v) BSA and washed again. The enzymatic substrate, p-nitrophenyl phosphate (pNPP) in 0.1 M diethanolamine-HCl (pH 9.8) was added, and the absorbance signal was measured at 405 nm after a 1 h incubation at 37 °C.

### Statistical analysis

Statistical analyses of HI data and ELISA results were performed using Mann–Whitney Rank-Sum test and T test (ELISA) in Sigma Plot software. *p*-values < 0.05 were defined as significant.

## Results

### Recombinant hemagglutinin-S·Tag fusion proteins and S·Protein variants are produced in planta

Trimeric hemagglutinin containing S·Tag and S·Protein variants were designed and expressed in planta, to generate influenza hemagglutinin oligomers based on the specific interaction of bovine S·Protein and the S·Tag (Figure 1). We fused the S·Tag (15 amino acid in length) c-terminally to the artificial trimerization domain (GCN4-pII) of the H5 hemagglutinin. This trimerization domain was proven to stabilize influenza hemagglutinin as trimers in planta [10]. An ubiquitous plant promoter (CaMV35S), a signal peptide and an endoplasmic reticulum (ER) retention signal (KDEL) allow for the production and accumulation of trimers in the ER of leaf cells after transient expression (Figure 2). The accumulation of such trimers has been analyzed by Western blot via the c-myc tag after separation of plant crude extracts at denaturing conditions in a SDS gel.

**Figure 3:**
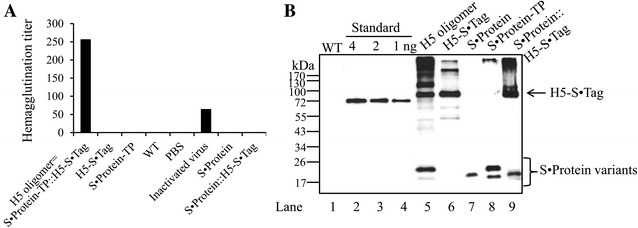
**Hemagglutinin oligomers from plants.** **A** Hemagglutination titers of plant extracts and inactivated virus rg A/swan/Germany/R65/2006(H5N1). *WT* wild-type *N. benthamiana*; *PBS* phosphate-buffered saline. **B** Hemagglutinin derivatives and S·Protein derivatives (30 µg total soluble protein/land) in plant extracts analyzed by anti-c-myc tag Western blot. Standard: anti-TNFalpha-nanobody-ELP [34]; S·Protein::H5-S·Tag: co-expression; H5 oligomer: S·Protein-TP::H5-S·Tag: co-expression.

A band corresponding to H5-S·Tag could be identified (Figure 3B, H5-S·Tag, lane 6).

We constructed an expression vector, that allows for the production of potentially multimeric wild-type S·Protein [23] in the plant ER (Figure 2) and performed transient expression experiments in *N. benthamiana* leaves. The analysis of crude extracts of these leaves by Western blot after separation at denaturating conditions revealed a major band that corresponds to a S·Protein monomer (Figure 3B, S·Protein, lane 7).

H5-S·Tag and S·Protein constructs were co-expressed in the ER of leaf cells. After co-infiltration of *N. benthamiana* with the appropriate *Agrobacterium* strains, leaf crude extracts were analyzed by Western blot after separation at denaturating conditions. Two major bands, reflecting H5-S·Tag and S·Protein, each corresponding in size to the molecular weights of the single expressed proteins were detected (Figure 3B, S·Protein::S·Tag, lane 9).

To multimerize the wild-type S·Protein, trimerization (GCN4-pII), dimerization (GCN4 wild-type) domains [9], or a tail piece of mouse IgM antibody that forms disulfide bonds via its cysteine residues were fused to the wild-type S·Protein C-terminally (Figure 2). Resulting recombinant proteins were S·Protein-pII, S·Protein-GCN4, and S·Protein-TP, respectively (Figure 2). Expression of these single recombinant S·Protein variants and co-expression with trimeric H5-S·Tag was always confirmed by Western blot analyses presented partially in Figure 3B and in Additional file 1 and summarized in Tables 1 and 2. Notably, hemagglutinin, S·Protein and the TP element contain five [24], one [25], and one [14] N-glycosylation sites, respectively. This influences the mobility in SDS gels and therefore, higher molecular weights in appearance will be detected (Figure 3B, Additional file 1). In general, all recombinant proteins were expressed in plants.

**Table 1:** **Expression and functionality profiles of recombinant influenza hemagglutinin and S·Protein variants**

Proteins	Protein expression confirmed by Western blot	Hemagglutination unit (HAU)	
		Plant extracts containing each single recombinant protein	Combination with H5-S·Tag in vitro
H5-S·Tag	(+)	0	
S·Protein	(+)	0	0
S·Protein-pII	(+)	0	0
S·Protein-GCN4	(+)	0	0
S·Protein-TP	(+)	0	0

Each single protein was transiently expressed in plants. The expression of proteins was confirmed by Western blot. Plant extracts containing S·Protein variants were combined with the H5-S·Tag containing plant extract in vitro. Mixtures were rotated at 4 °C for 1 h. The oligomer formation of all variants was investigated by hemagglutination assay. (+): expression of a single protein confirmed by Western blot.

**Table 2:** **Expression and functionality profiles of recombinant influenza hemagglutinin and S·Protein variants**

Proteins	Protein expression confirmed by Western blot	Hemagglutination unit (HAU)
		Co-expression with H5-S·Tag in plants in vivo
S·Protein	(++)	4
S·Protein-pII	(++)	0
S·Protein-GCN4	(++)	2
S·Protein-TP	(++)	256

Two proteins (one of the S·Protein variants and H5-S·Tag) were co-expressed transiently in plants. The expression of both proteins (++) was confirmed by Western blot. The oligomer formation of all variants was investigated by hemagglutination assay.

### Screening for an optimal hemagglutinin oligomerization tool

The hemagglutination assay is based on the ability of influenza hemagglutinin to bind sialic acid receptors presented on the surface of chicken red blood cells. Cross-linkages between influenza hemagglutinin and red blood cell cause the formation of a lattice called hemagglutination. In the hemagglutinin oligomers (Figure 1), cross-linkages between hemagglutinin trimers were already built via S·Protein and S·Tag interaction, while influenza trimers lack these linkages. Therefore, when hemagglutinin oligomers were mixed with given amount of red blood cells, high hemagglutination titers will be expected compared with those of trimeric hemagglutinin. This assay was used to screen for formation of hemagglutinin oligomers in plant crude extracts. The hemagglutination titers caused by the plant crude extracts containing single H5-S·Tag or S·Protein, as well as co-expressed proteins (H5-S·Tag and S·Protein) were all very low (Figure 3A; Table 2). We hypothesized that the wild-type S·Protein was not being sufficiently multimerized. Therefore, we fused different oligomerization motifs, such as the trimerization motif GCN4-pII, the dimerization motif GCN4 wild-type [9] and a tailpiece (TP) element of mouse IgM to the c-terminal end of the S·Protein sequence to multimerize the S·Protein. TP elements are responsible for the interchain connections between constant parts of single IgM chains to penta- or hexamers via disulfide bridges [14] (Figures 1 and 2). The principal plant expression constructs coding for S·Protein-pII, S·Protein-GCN4 and S·Protein-TP are shown in Figure 2. The plant expression has been tested by a transient assay in *N. benthamiana.* Each single crude extract was analyzed by a hemagglutination assay but no hemagglutination activity could be measured (Table 1). These S·Protein variants (S·Protein-pII, S·Protein-GCN4, and S·Protein-TP, respectively), were co-expressed with H5-S·Tag. In parallel, extracts containing the single S·Protein variants, were each mixed in vitro with extracts containing H5-S·Tag. These extracts respectively mixtures were tested in hemagglutination assays. Only the variant co-expression of S·Protein-TP with H5-S·Tag (named as H5 oligomers) was found to cause a very high hemagglutination titer of 256 (Figure 3A; Table 2). All other variants, including the S·Protein-TP with H5-S·Tag mixtures conducted in vitro, did not cause increased hemagglutination titers (Tables 1 and 2). The analysis of H5 oligomer extracts, H5-S·Tag extracts and S·Protein-TP extracts by Western blot revealed major bands of expected sizes (Figure 3B, lanes 5, 6 and 8). Notably, the co-expression of H5-S·Tag and S·Protein-TP had no significant effect on the accumulation of hemagglutinin (Figure 3B). Hemagglutinin was accumulated in plants with around 0.2% of total soluble proteins estimated by Western blot (data not shown).

### Formation of H5 oligomers in planta but not in vitro

**Figure 4:**
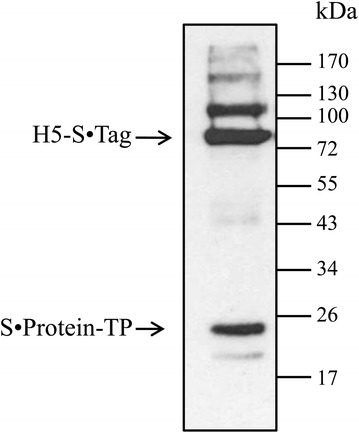
**Integration of S·Protein-TP in the H5 oligomers.** H5-S·Tag proteins were purified from plant extracts containing co-expressed proteins: S·Protein-TP (without His tag) and H5-S·Tag by IMAC. The purified product was analyzed by anti-c-myc tag Western blot. The presence of S·Protein-TP band shows the binding between S·Protein-TP and H5-S·Tag to form H5 oligomers.

**Figure 5:**
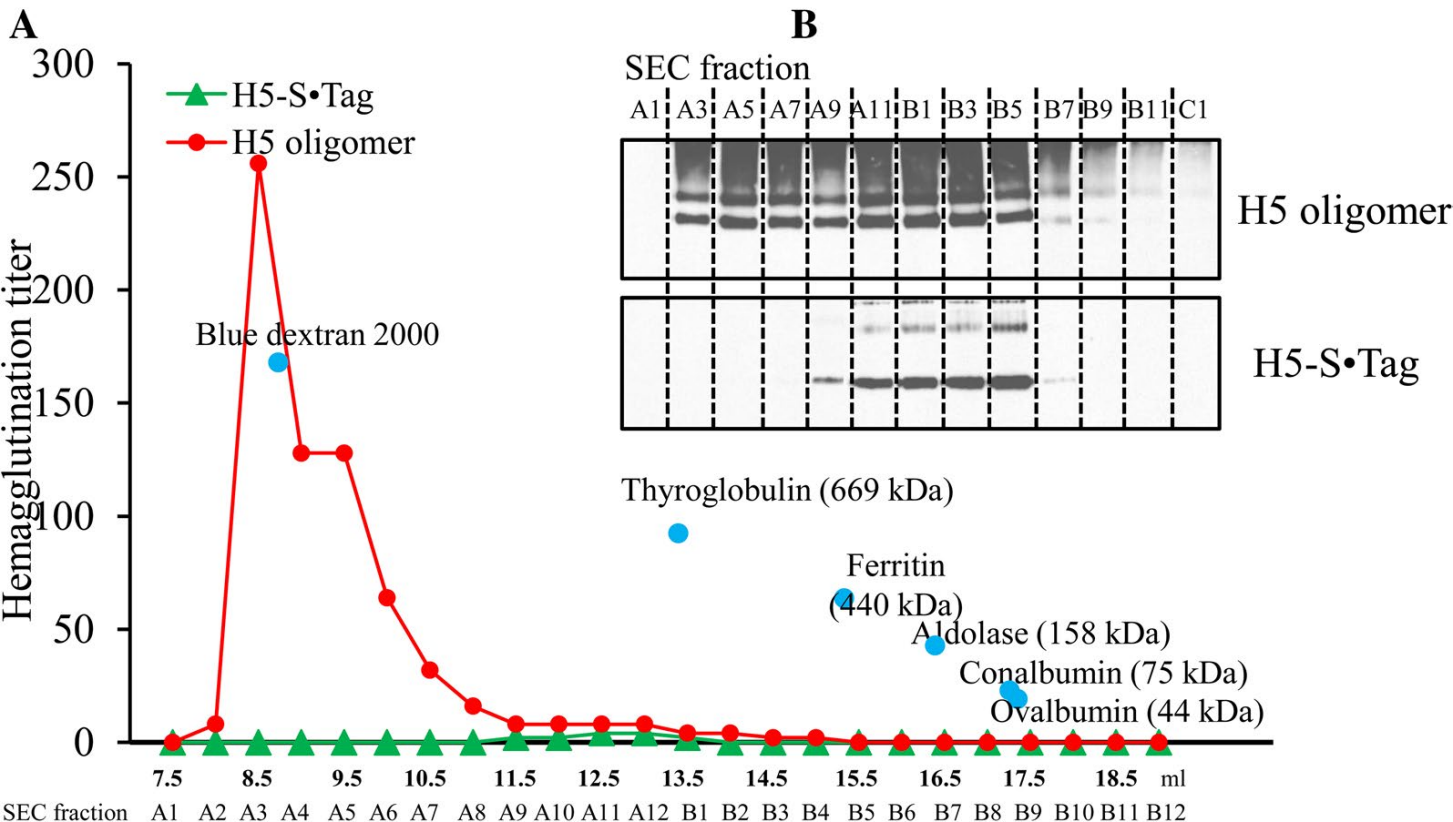
**Oligomeric H5 formation, as demonstrated by size exclusion chromatography, hemagglutination and Western blot analyses.** IMAC-purified H5 oligomers or H5-S·Tag (each 34 µg in 0.5 mL) were separated on Superose™ 6 increase 10/300 GL, and the fractions were analyzed by hemagglutination assay (**A**) and Western blot (**B**).

The H5-S·Tag protein contains a His tag, whereas S·Protein-TP does not contain this tag. Both proteins contain the c-myc tag (Figure 2). Therefore, we could analyze by co-purification if the H5-S·Tag protein interacts with S·Protein-TP. For this purpose H5 oligomers were purified by immobilized metal affinity chromatography (IMAC). The purified products were then analyzed by Western blot. Both H5-S·Tag and S·Protein-TP (without 6 × His) were detected (Figure 4). The co-purification of the S·Protein-TP indicated specific interaction of this protein with the H5-S·Tag. The further characterization of the potential oligomers was done at native conditions, thus keeping the oligomeric structure intact. Purified H5 oligomers and purified H5-S·Tag were separated by size exclusion chromatography (SEC) and the hemagglutination titer of every fraction was estimated. High hemagglutination titers were observed in fractions A3 to A8 of H5 oligomers. The highest molecular weight (fraction A3, 2000 kDa) corresponds to the highest hemagglutination titer (Figure 5A). The analysis of H5-S·Tag by SEC did not show high molecular weights or high hemagglutination titers (Figure 5A). The fractions of H5 oligomers were separated by SDS-PAGE and analyzed in parallel with fractions of H5-S·Tag (without S·Protein-TP) by Western blot. This analysis showed that very high molecular weight hemagglutinins (> 700–2000 kDa, fractions A7–A3) were exclusively achieved in H5 oligomer extracts after co-expression of H5-S·Tag and S·Protein-TP, but not after expression of a single component (Figures 5A and B). This result indicated that the H5 oligomers were a mixture of oligomers which are made from different numbers of H5-S·Tag and S·Protein-TP incorporated into the complexes (Figure 1). Based on calculated size of trimeric H5-S·Tag (200 kDa), and the size of H5 oligomer determined by SEC, the number of trimeric H5-S·Tag integrated in H5 oligomers ranged from 1 to 8.

The protein expression of each S-tagged H5 trimers and S·Protein-TP in different plants and the in vitro combination of the extracts caused hemagglutination titers of 0 (Table 1), although production of the different components at the expected molecular weights was shown by Western blot (Figure 5B, summarized in Tables 1 and 2). The low hemagglutination titers reflect that hemagglutinin oligomers were not formed in vitro. As shown in previous studies, functionally active S·Protein was only yielded in the presence of the S·Tag. Obviously, the S·Tag serves as a template for proper folding of S·Protein [12, 13, 26]. We suppose, that S·Protein-TP is expressed in an inactive form in the absence of H5-S·Tag and therefore it does not interact with H5-S·Tag in vitro. Exclusively, the co-expression of H5-S·Tag and S·Protein-TP caused high hemagglutination titers as well as very high molecular weights by production in planta in the ER, thus allowing folding and oligomerization by closure of disulfide bridges. This variant presents a new and innovative way to generate functional H5 oligomers in planta.

### H5 oligomers are highly immunogenic

**Figure 6:**
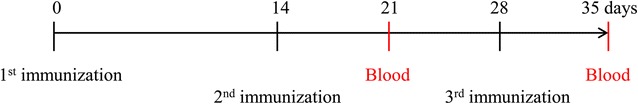
**Mouse immunization and bleeding schedule.** Each mouse was immunized either with crude extracts containing 0.1 µg (H5 oligomer or H5-S·Tag) formulated with the Emulsigen^®^-D adjuvant at 20% final concentration (two experimental groups) or with *N. benthamiana* wild type leaf extracts or S·Protein-TP leaf extracts formulated with the Emulsigen^®^-D adjuvant at 20% final concentration (negative control groups). Comparable leaf protein amounts were in applied to each mouse. Twelve mice per group were vaccinated with the formulated vaccines at days 0, 14 and 28. One week after the 2^nd^ and 3^rd^ immunizations, mice were bled via the retro-orbital sinus.

**Figure 7:**
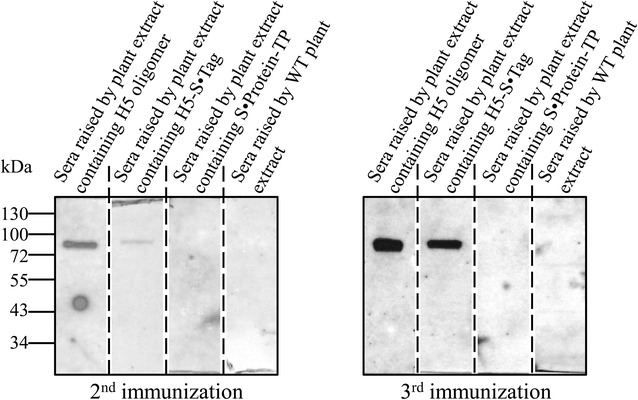
**Strong and specific immune responses induced by H5 oligomers compared to trimers in mice analyzed by Western blot.** H5 specific binding of antibodies from mixtures of 10 sera raised against corresponding plant extracts as demonstrated by Western blot. Identical serum dilutions (200 times dilution) of each group were used to recognize 10 ng of the H5 hemagglutinin.

**Figure 8:**
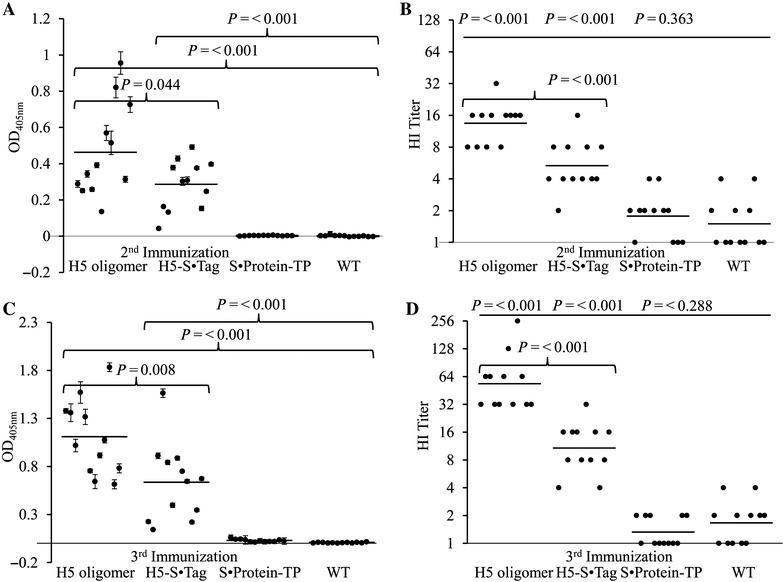
**Immunological characterization of H5 oligomer, H5-S·Tag and S·Protein-TP extracts compared to wild-type extracts.** **A**, **B** Antibody responses after the 2^nd^ immunization. Measurement of antibody responses raised by injection of different extracts into mice after two immunizations by indirect ELISA (**A**) and hemagglutination inhibition assay (**B**). **C**, **D** Antibody responses after the 3^rd^ immunization. Measurement of antibody responses raised by injection of different extracts into mice after three immunizations by indirect ELISA (**C**) and hemagglutination inhibition assay (**D**). *P*-value. A single dot represents the ELISA result from a single serum sample measured in five parallels. Standard deviations are given. Bars are the mean of each test group. Measurement of hemagglutination inhibition titers of sera raised against the extracts mentioned above. A single dot represents the hemagglutinin titer of the single serum sample, and bars are the geometric mean titre of each test group.

The immunogenicity of the H5 oligomers was tested in comparison to the immunogenicity of the components H5-S·Tag and S·Protein-TP by immunization of mice. Each 12 C57Bl6/J mice were immunized with either wild-type plant crude extracts or crude extracts containing H5 oligomers, H5·S-Tag trimers or S·Protein-TP, respectively (Figure 6). Crude extracts were chosen, because animal vaccine development should fit into the economical demands of mass immunizations in chicken, especially in terms of minimized down-stream processing. The resulting antibody-dependent humoral immune responses were firstly tested against purified hemagglutinin H5 in Western blot (Figure 7). Specific antibodies against purified hemagglutinin H5 have been detected after 2 and 3 immunizations by plant extracts containing H5 oligomers and, to a lower extend, after immunizations by H5-S·Tag. After 3 immunizations, much stronger bands were visible. The immune responses in the sera of each 12 mice per group have been measured against purified hemagglutinin H5 by an indirect ELISA. Whereas no immune response against hemagglutinin H5 was, as expected, detected after immunization with crude extracts containing S·Protein-TP or wild-type crude extracts, specific binding to purified hemagglutinin H5 was measured after immunization with H5 oligomer and H5-S·Tag crude extracts (Figures 8A and C). The immunogenicity of H5 oligomers was significantly higher, especially after three immunizations with a *p* value of 0.008 (Figure 8C). Hemagglutination inhibition (HI) assays were performed to measure, if neutralizing antibodies could be induced. Because the A/duck/Viet Nam/TG24-01/2005 (H5N1) virus was unavailable in an inactivated form, the heterologous inactivated virus strain rg A/swan/Germany/R65/2006(H5N1) was used instead for the HI assay. The deduced hemagglutinin amino acid sequence similarity of both strains is 96%. HI assays showed that neutralizing antibodies inhibiting hemagglutination were produced in mice by immunization with H5 oligomer crude extracts and with H5-S·Tag crude extracts (vaccine groups) after 2nd immunization, and their HI geometric mean titres (HI GMTs) were 13.5 and 5.3, respectively (Figure 8B). The neutralizing antibody response was significantly better after immunization two times with H5 oligomer extracts compared to the sera of mice immunized two times with H5-S·Tag trimer extracts (*p* < 0.001, Figure 8B). This comparison shows the neutralization enhancing effect of oligomerization. In mice vaccinated two times with wild-type plant crude extracts and S·Protein-TP crude extracts (negative control groups), most of the mouse sera caused low HI titres. The HI GMTs of these groups were 1.8 and 1.5, respectively. These HI GMTs were much lower than those of sera from mice vaccinated with H5 oligomer and H5-S·tag (trimer) crude extracts. A fourfold increase in HI GMTs was observed in sera from mice vaccinated with H5 oligomer crude extracts compared to sera from mice immunized with S·Protein-TP crude extracts and wildtype crude extracts. A fourfold increase in HI titres is associated with two-fold decrease in the risk of infection [27] and defined as seroconversion [28]. Following the third immunization, the HI GMTs of vaccine groups were higher than after the second immunization, especially in the sera derived from mice vaccinated with H5 oligomer crude extracts (Figures 8B–D). The HI GMTs of these mice were 53.8 and 10.7, while the negative control sera HI titres were as low as 1.3 and 1.7. Again, neutralizing antibody titres induced by the H5 oligomer crude extracts were significantly higher than those induced by H5-S·Tag crude extracts (Figure 8D). A fourfold increase in HI titres was now observed in the both vaccine groups compared to sera from mice immunized with S·Protein-TP crude extracts and wild-type crude extracts.

We conclude that plant crude extracts containing H5 oligomers are more immunogenic than the trimeric H5-S·Tag containing extracts as measured by an indirect ELISA. H5 oligomers induce neutralizing antibodies to a significantly higher extend compared to trimeric H5-S·Tag vaccines. The stability of H5 derivatives in crude extracts is important for the feasibility of the concept. The immunogenic extracts were stored at 4 °C for 1 week without loss of antigen content, as revealed by Western blot and hemagglutination titer (see Additional file 2).

## Discussion

The transient expression system has emerged as an alternative platform to produce influenza subunit vaccine candidates because of its capacity to be easily scaled up and because of the rapid production process [7, 29]. The method of infiltration of plant leaves with *Agrobacteria* harboring the transgene is a robust and technologically simple method [30]. Two major approaches have been developed to efficiently express influenza vaccine candidates: expression of hemagglutinin ectodomains from swine flu H1N1 strains [31] or from avian flu H5N1 strains [10] and production of enveloped hemagglutinins as VLPs [7, 8, 29]. All plant-made hemagglutinin ectodomains tested to date were expressed as soluble monomeric [10, 32] as well as trimeric proteins stabilized by trimeric motifs [10, 31]. Artificially trimerized hemagglutinin ectodomains without S·Tag fusion, mimicking hemagglutinin homotrimers on the viral surface, enhanced immunogenicity, induced neutralizing antibodies [10] and reduced the necessary vaccine doses [31]. In the actual study, trimeric hemagglutinin fused with S·Tag (H5-S·Tag) in crude extracts was confirmed again to induce neutralizing antibodies (Figures 8B and D). Here, we present a strategy to produce hemagglutinin trimer-based antigen oligomers. This concept is based on the specific interaction of bovine S·Protein and the S·Tag, both of them are cleavage products of ribonuclease A by the proteinase subtilisin [12]. The trimerization by a specific domain (GCN4-pII motif [9, 10]) served as a founding structure of putative oligomers to design basic structures resembling native hemagglutinin homotrimers (see above). These hemagglutinin trimers should be further multimerized by interaction of an S·Tag fused to hemagglutinin with S·Proteins via co-expression of both proteins in the ER of plant cells. S·Proteins themselves are expected to consist of a mixture of monomeric, dimeric, trimeric and tetrameric proteins (or even more), as could be concluded from a previous study using ribonuclease A [33] (reviewed by [23]). Obviously, this was not sufficient to produce oligomeric H5 in the ER of plant cells (Tables 1 and 2). The fusion of dimerization and trimerization domains to the C-terminus of the S·Proteins and the subsequent co-expression in the plant cell ER (Figures 3A and B) also did not induce oligomers in high concentrations as could be concluded from low hemagglutination titers (Figure 3A; Tables 1 and 2). However, the plant extracts achieved after co-expression of H5-S·Tag and S·Protein-TP (containing the TP sequence at the c terminus) showed significantly increased hemagglutination titers compared to hemaglutination titers induced by H5-S·Tag or S·Protein-TP, respectively (Figures 3A and B; Table 2). This could be explained by the different structures of dimerization or, trimerization motives and disulfide bonds. The dimerization and trimerization domains are parallel, coiled coil structures [9] which can force their fusion partners to dimerize or trimerize in their fixed angles. However, the disulfide bonds formed by cysteine residues in the TP elements are flexible joins allowing S·Protein-TPs to fold correctly into an active form and allowing S·Protein-TPs to bend, twist, and flex into an optimal position to bind H5-S·Tag to finally form oligomers.

We conclude, that large oligomers were built. When a tail piece sequence was fused to wild-type S·Protein, monomeric, dimeric, trimeric or tetrameric S·Protein-TP joined with themselves or with others to generate multimerized S·Protein-TP via disulfide bonds. The plant ER, containing protein disulphide isomerases, is the perfect compartment for these processes. The resulting multimeric S·Protein-TPs have multiple valences to bind trimeric H5-S·Tag proteins to form H5 oligomers. This process is partially presented in Figure 1. In fact, H5 oligomers are a mixture of different numbers of H5-S·Tag and S·Protein-TP incorporated into the complexes (Figures 1, 4 and 5). Larger complexes cause higher hemagglutination titers (Figure 5). These large complexes cause improved neutralizing immune responses as shown by higher hemagglutination inhibition titers (Figure 8B and D). The low *p* values (*p* ≤ 0.001) comparing the HI titers after immunizations with either H5 oligomer or H5-S·Tag after 2 and 3 immunizations document the significance of these differences. Other crucial prerequisites for the successful development of a veterinary vaccine against avian flu are speed and practicability. *Agrobacterium* infiltration systems allow for the production of large amounts of proteins only a few days after finishing the cloning procedure, and thus, this concept generally shares speed and convenience [34]. The lack of down-stream processing effort also fits into the general timeline demands for flu vaccines [19]. Downstream cost for recombinant expression systems can represent up to 80% of the overall processing cost [35]. These high downstream costs are a major bottleneck limiting the commercial production of plant-based pharmaceuticals [36]. Thus, the successful use of crude extracts for immunization as performed in the actual study can significantly lower down-stream cost. This is essentially important for veterinary vaccines, where cost have to be low to fit into economical parameters of animal-based production [37]. The principles shown and discussed here will generally allow for the development of low cost vaccines with unlimited scalability as precautions for veterinary immunotherapies [5]. In the actual paper, we use a synthetic biology approach to combine different principles of protein–protein interaction from different organisms to design an innovative vaccine concept in plants [38].

## Additional files

**Additional file 1.** **Expression of recombinant proteins in plants. Hemagglutinin derivatives and S·Protein derivatives (30 µg total soluble protein/lane) in plant extracts analyzed by anti-c-myc tag Western blot.** S·Protein-pII::H5-S·Tag: co-expression; S·Protein-GCN4::H5-S·Tag: co-expression; S·Protein-pII, S·Protein-GCN4, and H5-S·Tag: single expression; WT: wild-type *N. benthamiana*.

**Additional file 2.** **Stability of H5 oligomers in plant crude extracts.** (A) Western blot analysis of each 20 µL of H5 oligomers stored as crude extracts on ice. (B) H5 oligomer stability after storage of crude extracts on ice measured by hemagglutination in comparison to inactivated virus and negative control (PBS).
