# Supplementary figures and images for: Neutralizing immune responses induced by oligomeric H5N1-hemagglutinins from plants
Source: Vet Res. 2017 Sep 20;48:53. doi: 10.1186/s13567-017-0458-x (PMC5607582; doi:10.1186/s13567-017-0458-x)

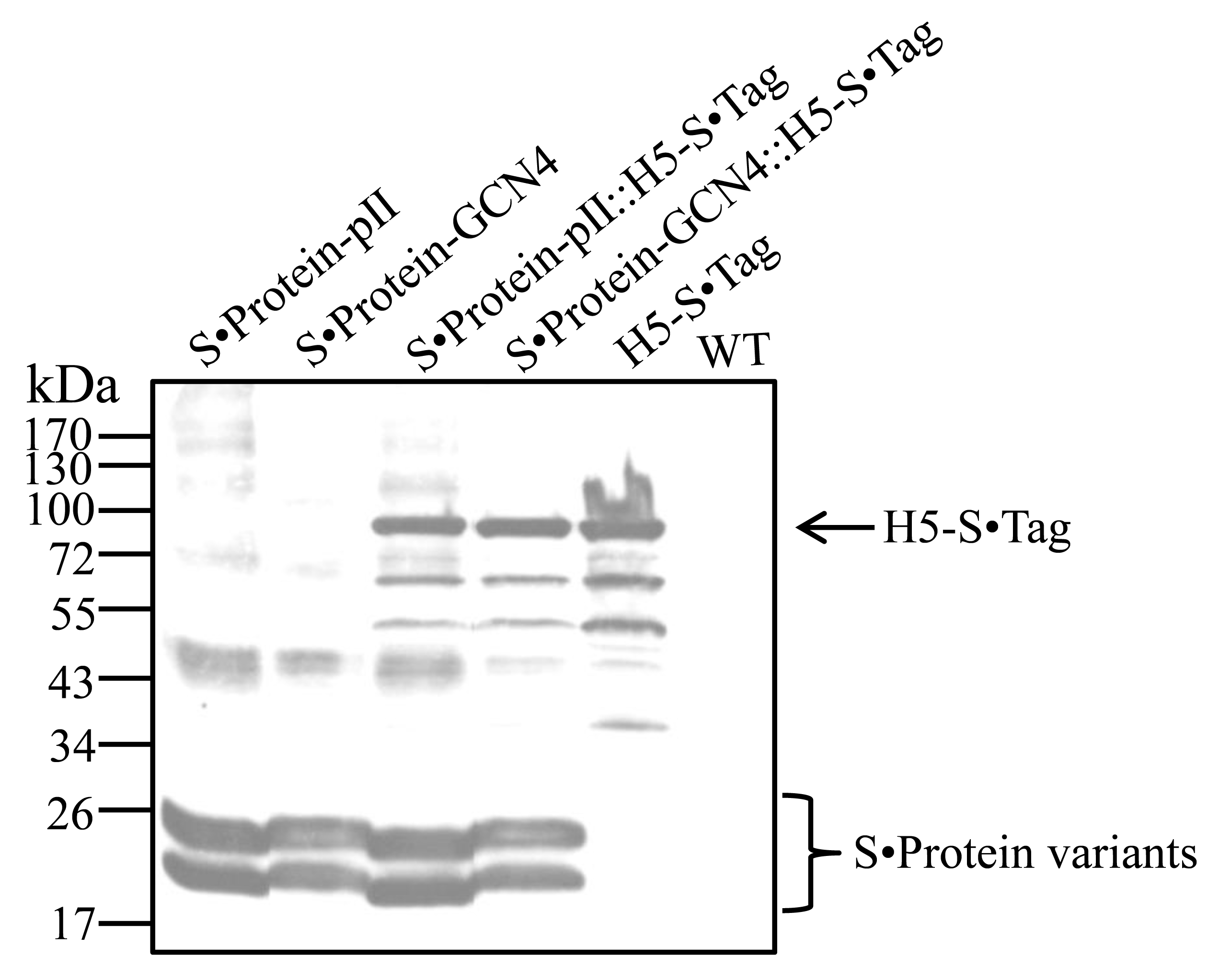

Supplement: Supplementary file 1 — Additional file 1. Expression of recombinant proteins in plants. Hemagglutinin derivatives and S·Protein derivatives (30 µg total soluble protein/lane) in plant extracts analyzed by anti-c-myc tag Western blot. S·Protein-pII::H5-S·Tag: co-expression; S·Protein-GCN4::H5-S·Tag: co-expression; S·Protein-pII, S·Protein-GCN4, and H5-S·Tag: single expression; WT: wild-type N. benthamiana. [file 13567_2017_458_MOESM1_ESM.tif]

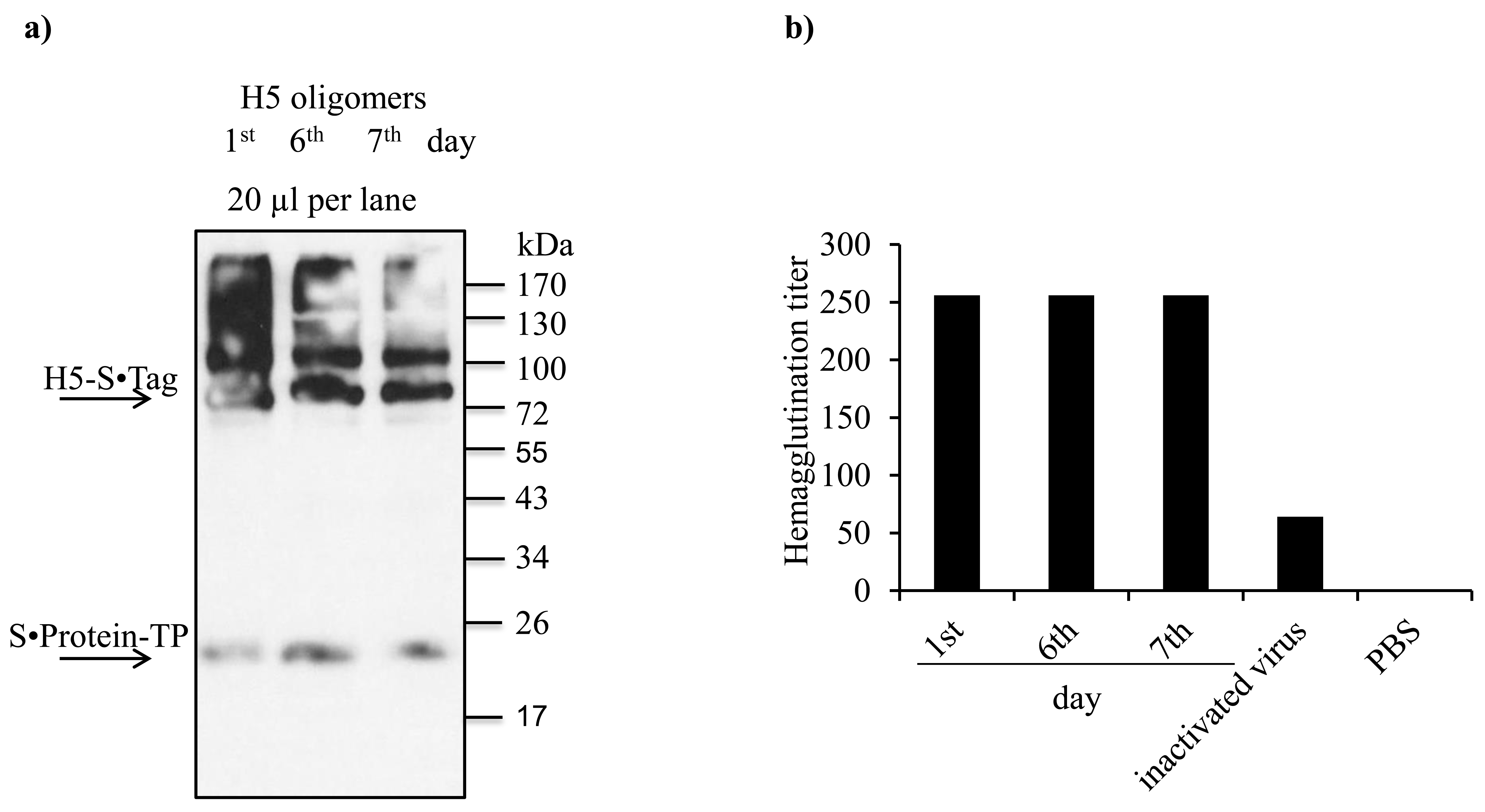

Supplement: Supplementary file 2 — Additional file 2. Stability of H5 oligomers in plant crude extracts. (A) Western blot analysis of each 20 µL of H5 oligomers stored as crude extracts on ice. (B) H5 oligomer stability after storage of crude extracts on ice measured by hemagglutination in comparison to inactivated virus and negative control (PBS). [file 13567_2017_458_MOESM2_ESM.tif]
